# Hepatic myofibroblasts derived from *Schistosoma mansoni*-infected mice are a source of IL-5 and eotaxin: controls of eosinophil populations in vitro

**DOI:** 10.1186/s13071-015-1197-3

**Published:** 2015-11-09

**Authors:** Ligia Almeida Paiva, Camila Brand, Christianne Bandeira-Melo, Patricia Torres Bozza, Marcia Cury El-Cheikh, Patricia Martins Silva, Radovan Borojevic, Sandra Aurora Chavez Perez

**Affiliations:** Laboratory of Immunopharmacology, Institute Oswaldo Cruz, FIOCRUZ, Rio de Janeiro, Brazil; Institute of Biomedical Sciences, Federal University of Rio de Janeiro, Rio de Janeiro, Brazil; Institute of Biophysics Carlos Chagas Filho, Federal University of Rio de Janeiro, Rio de Janeiro, Brazil; Laboratory of Inflammation, Institute Oswaldo Cruz, FIOCRUZ, Rio de Janeiro, Brazil; Faculty of Medicine / FASE, Petrópolis, RJ Brazil; Farmanguinhos, FIOCRUZ, Rio de Janeiro, Brazil

**Keywords:** Liver myofibroblasts, IL-5, Eotaxin, *Schistosoma mansoni*, Eosinophil granulocyte

## Abstract

**Background:**

Hepatic myofibroblasts are relevant for pathogenesis of *S. mansoni* infection. In normal liver, these perisinusoidal cells are quiescent, express the lipocyte phenotype, and are located in the Disse’s space, being the major site of vitamin A storage. When activated, they convert to myofibroblasts and contribute to granulomatous and diffuse liver fibrosis. In the present work, we observed that myofibroblasts obtained from granulomatous periovular inflammatory reactions in schistosome-infected mice (GR-MF) produce *in vitro* immunomodulatory cytokines for eosinophil activation: IL-5 and eotaxin.

**Methods and results:**

The secretory activity of GR-MF was detected after TGF-β and IL-13 stimulation using 2D and 3D cell culture systems. In a mixed co-culture system using GR-MF with hematopoietic bone marrow cells from infected mice, we observed eosinophil survival that was dependent upon IL-5 and eotaxin, since antibodies against this cytokines decreased eosinophil population, as measured by eosinophil peroxidase activity.

**Conclusion:**

These results indicate that GR-MF may contribute to maintenance of local eosinophilia in schistosomal hepatic granulomas, and can function as immunoregulatory cells, besides their role in production of fibrosis.

**Electronic supplementary material:**

The online version of this article (doi:10.1186/s13071-015-1197-3) contains supplementary material, which is available to authorized users.

## Background

Schistosomiasis is one of the major endemic parasitic diseases in the world. *Schistosoma mansoni, S. haematobium*, and *S. japonicum* are the species of major medical relevance for humans [1, 2]. The host tissue injuries mostly occur in response to schistosome eggs deposited in the intestinal venous circulation, from which they reach the intestinal lumen, and are subsequently eliminated. Alternatively, they are brought into liver by the mesenteric and portal circulations, being entrapped in the hepatic tissue, where they remain, eliciting formation of inflammatory granulomas, which are a response to eggs and myracidum-produced secreta that diffuse into the adjacent tissues [3, 4]. Consequently, one of the major features of schistosomiasis is a chronic liver inflammation and fibrosis.

In the acute phase of infection, the systemic and the periovular granulomatous reactions represent a classical Th2 response, orchestrated by CD4^+^ T cells, with a rich infiltration of eosinophils and macrophages. During the chronic phase, installation of an immune response controlled by Th1, Th17 and T regulatory cells and reduction of the granuloma size are observed, in parallel to increased fibrosis [3, 5–7].

Similar to other parasites infections, schistosomiasis is hyperergic in the acute phase of the disease, with high levels of inflammatory mediators including the stimulators of eosinophil production and mobilization. In this phase, eosinophils are essentially produced in the bone marrow, where up to 50 % of the myeloid cell population may be involved in eosinopoiesis. The circulating ones are readily mobilized into the infected tissues, and only immature eosinophils are found in the blood [8]. Their production is essentially dependent upon IL-5 [9].

In the chronic phase of schistosomiasis, a moderate increase of eosinophil precursors in bone marrow is associated with a mild blood eosinophilia. The intense tissue eosinophilia is dependent upon the peripheral production of myeloid cells. This occurs essentially in sites where schistosome eggs are deposited: within the mesenteric vessels of the intestinal wall and in the adjacent tissues, as well as within the intrahepatic portal venous vascular system in which eggs are trapped [7].

In the abdominal cavity of mice with chronic schistosomiasis, experimental induction of eosinopoiesis was correlated with mobilization of macrophages [10]. Alternatively, it was associated with coelom-associated lympho-myeloid tissue (milky spots), which occasionally grew in large polypoid structures with rich eosino- and plasmacytogenesis [11, 12]. Within these structures, as well as in the omentum, myelopoiesis was associated with macrophages and with the adipose tissue stroma, which produced the major hemopoietins (GM-CSF, M-CSF, G-CSF, IL-5) as well as SDF-1α, potentially involved in homing of circulating progenitors. Notably, the normal stroma produced LIF, while schistosome-infected mice stroma produced SCF. The latter one may be associated with induction of an intense proliferation of myeloid cells [13].

Similar to the abdominal cavity, in chronic schistosomiasis a hepatic eosinopoiesis was described in two scenarios. Eosinopoietic foci were observed within the sinusoids, in close contact with hypertrophic Kupffer-cells but not with perisinusoidal cells. The eosinophil progenitors and their maturation within these foci were synchronized, suggesting a local clonogenic growth of exogenous progenitor cells retained in the liver tissue [14]. Conversely, groups of eosinophil granulocytes in different stages of maturation were observed within the periovular granulomas, indicating a continuous active input of myeloid progenitors. They were in close contact with macrophages and with extracellular matrix containing collagen and proteoglycans [15]. Stimulation of eosinopoiesis was traced to factors produced by macrophages, but production of IL-5 could not be demonstrated [8, 16]. Similar to mesenteric stroma, connective tissue cells obtained from granulomas, which are at least in part derived from perisinusoidal cells, are a rich source of hemopoietins [17]. Moreover, under stimulation by TNFα, the GR-MF stroma produces SCF that sustains proliferation of myeloid cells [18]. Hepatic stellate cells are now considered to belong to the group of mesenchymal perivascular stroma cells, which participate in tissue reaction to injury [19]. When activated, they are also involved in regulation of hepatic inflammation and immunity through the expression of inflammatory and immune regulatory molecules [20].

In the present study, we have addressed the question of whether granuloma-derived myofibroblasts stimulated with TGF-β and IL-13, the pro-fibrogenic cytokines, can promote and sustain production and survival of eosinophil granulocytes. We have focused our attention on IL-5 and eotaxin production in 2D and 3D cell culture systems. We propose that GR-MF have a major role in production, maintenance and activation of eosinophil granulocytes in chronic inflammatory tissue reactions to parasites.

## Methods

### Ethics statement

This study was carried out in accordance with the recommendations of the Guide for Care and Use of Laboratory Animals of the Brazilian National Council of Animal Experimentation. The protocols were approved by the Oswaldo Cruz Foundation Animal Welfare Committee (CEUA LW-32/12).

### Histological preparations

For histological analyses, animals were sacrificed after 90 days of infection. Livers were removed, cut into 0.5 mm thick slices, washed in cold saline and fixed in Bouin’s fixative. After 6 h of fixation, specimens were dehydrated in alcohol, and embedded in paraffin. Sections of 5 μm were obtained and stained with Hematoxylin-eosin and Masson’s trichrome staining.

For Masson’s Trichrome stain, briefly, slides were placed in Bouin’s solution for 1 h at 56 °C, then stained with Weigert’s hematoxylin (10 min; Sigma-Aldrich, St. Louis, MO), followed by Biebrich scarlet acid fuchsin (5 min; Sigma-Aldrich), phosphomolybdic/phosphotungstic acid solution (10 min; Sigma-Aldrich), and aniline blue (5 min; Sigma-Aldrich).

For α-smooth muscle actin (α-SMA) immunofluorescence, paraffin-embedded sections were dewaxed and hydrated and washed several times in PBS and further treated with 50nM NH_4_Cl (30 min), 0.05 % saponin/0.1 M PBS (30 min) and 0.05 % gelatin/0.05 % saponin/PBS (30 min). Cells were incubated with human monoclonal anti-SMA (Sigma-Aldrich, Inc., St. Louis, MO) diluted 1:400 in 0.05 % gelatin/0,05%saponin/PBS as the primary antibody at 4 °C, overnight. Thereafter, sections were incubated with anti-human Alexa Fluor® 546 (Life Technologies, Inc.) diluted 1:1000. Sections were examined by confocal microscopy Olympus FV-1000.

### Isolation and purification of primary murine hepatic myofibroblasts derived from granulomas of schistosome-infected mice

C3H/HeN mice were obtained from the colony-breeding unit of the Oswaldo Cruz Foundation (Rio de Janeiro, Brazil). Mice were infected by 30–40 cercariae of *Schistosoma mansoni* (BH strain, Institute Oswaldo Cruz, RJ). Mice were killed 90 days after infection, periovular granulomas were isolated from mechanically homogenized liver tissue by sedimentation, and digested by collagenase 1A (1 mg/mL) (Sigma-Aldrich, Inc., St. Louis, MO). The harvested cells were seeded into 25 cm^2^ tissue culture flask in DMEM with 10 % fetal bovine serum (FBS). Cells were subcultured by trypsinization that eliminated the trypsin-resistant granuloma macrophages. After the fourth passage, a homogeneous population of myofibroblasts derived from hepatic granulomas were thus obtained (Additional file 1: Figure S1). These cells, named GR-MF, were fully described and characterized in previous studies [21–24].

### Treatments

GR-MF maintained in DMEM with 2 % FBS, were stimulated with TGF-β (Peprotech) (30 and 60 ng/mL) and IL-13 (Peprotech) (40 ng/mL). After 24 h, cells were collected for mRNA extraction, and supernatants were stored at -20 °C for quantification of IL-5, eotaxin and collagen.

### Three-dimensional (3D) culture of GR-MF

GR-MF monolayers were trypsinizated and cells were seeded in 1 % agarose- coated round-bottom 96-well plates. Cells were stimulated with TGF-β (30 ng/mL) and IL-13 (40 ng/mL). Spheres were observed and collected at day 4 for histological analysis, and supernatants were stored at -20 °C for quantification of IL-5, eotaxin and collagen. The diameters were measured using digital images.

### Histological preparations of spheroids

For histological analyses, spheroids were washed in cold saline and fixed in Bouin’s solution. After 4 h fixation, specimens were dehydrated in alcohol and embedded in paraffin. Sections of 5 μm were obtained and stained with Hematoxylin and Eosin (HE), and a Modified Sirius Red technique for collagen [25].

For α-SMA staining, paraffin-embedded sections were dewaxed and hydrated. After inhibition of endogenous peroxidase, sections were incubated for 1 h with 5 % BSA/0.1 % triton X-100/0.05 % tween-20/10 % normal goat serum/PBS followed by incubation of monoclonal anti α-SMA (1:400, Sigma-Aldrich, Inc., St. Louis, MO) overnight at 4 °C in a humid chamber. α-SMA was detected with a biotinylated anti mouse IgG (BA-4001, Vector Laboratories; Burlingame, CA, USA) and developed with avidin-peroxidase (1:50 in PBS, Sigma Immuno Chemicals, St. Louis, MO, E-8 8386), using diaminobenzidine as chromogen. Sections were counterstained with Harris’ hematoxylin. As negative controls, sections were incubated with non-immune mouse serum.

### mRNA extraction and real time reverse transcriptase-polymerase chain reaction (RT-PCR)

mRNA was extracted from 0.5 to 1 × 10^6^ cells using TRIzol® Reagent following standard protocols. Total RNA was dissolved in water, quantified and stored at -20 °C. cDNA was synthesized from 1 μg total RNA. RNA was primed with 0.5 μg oligo-(dT) primer, denatured (10 min at 64 °C) and cooled on ice. The following reagents were mixed and added: 10 mM of each deoxynucleoside triphosphate (dNTP), 6 μL 5X reverse transcriptase (RT) buffer, 0.1 mM DTT and 200 U of MLV-RT. Reaction was performed by incubation for 1 h at 37 °C.

During amplification, we used pre-designed TaqMan Gene Expression Assays (Applied Biosystems), according to the manufacturer’s instructions, for IL-5 (NCBI RefSeq: NM_010558.1), eotaxin (NCBI RefSeq: NM_011330.3) and GAPDH (NCBI RefSeq: NM_001289726.1). The qPCR reaction was performed with a final volume of 25 μL, containing 2μL of cDNA, 12.5 μL of master mix (Applied Biosystems), and 250 nmol primers and probe in optical 96-well plates. The fluorescence emission from each reaction was measured three times during the annealing/extension, and amplification plots were analyzed using software from Applied Biosystems. The gene expression data were normalized using GAPDH as housekeeping gene.

### Immunofluorescence analysis of GR-MF

GR-MF were seeded into 24-well culture plates at a concentration of 1 × 10^4^ cells/well, in 500 μL standard culture medium, and fixed after 2 days culture. Cells were washed with HBSS solution and fixed in 4 % paraformaldehyde in PBS buffer, pH 7.4 for 15 min. After fixation, single-cell preparations were washed several times in PBS and further treated with 50 nM NH_4_Cl (30 min), 0.05 % saponin/PBS (30 min) and 0.05 % gelatin/0.05%saponin/PBS (30 min). Cells were treated with primary antibody, rat monoclonal anti-CCL11/eotaxin (R&D Systems) 5 μg/mL diluted 0.05 % gelatin/0.05%saponin/PBS, at 4 °C, overnight. Thereafter, cells were treated with a biotinylated IgG goat anti-rat antibody (Vector Labs) at the dilution indicated by the manufacturer. Fluorescence labeling was performed with extravidinCy3 (Sigma-Aldrich, Inc., St. Louis, MO) diluted 1:600. In both protocols, cells were examined with an Olympus epifluorescence microscope, and images were acquired using the ImagePro program.

### IL-5 and eotaxin measurement

IL-5 and eotaxin in the supernatants of untreated and treated GR-MF were assayed by ELISA according to the manufacturer’s instructions (R&D Systems).

### Proliferation assay

GR-MF were seeded into 24-well culture plates and stimulated with TGF-β (30 and 60 ng/mL), diluted in DMEM with 2 % fetal bovine serum. After 20 h, we added 0.5 μCi/mL 3[H]-thymidine and incubated in 5 % CO_2_ at 37 °C for 4 h. Cells were washed three times with PBS and harvest with 1 mL 0.1 % Triton/PBS. The solubilized cell solution was added into 2 mL scintillation vials and counted in a scintillation counter (Beckman LS 6500).

### Collagen measurement

Collagen was quantified in supernatants of untreated and treated GR-MF using SircolKit (Biocolor, UK). The result was expressed as μg of collagen/mg total protein.

### Co-culture of GR-MF and hematopoietic bone marrow cells

GR-MF were plated on glass coverslips to form a monolayer. After 4–6 days, a cell fraction of nonadherent mononuclear bone marrow cells of schistosome infected C3H/HeN mice were added under GR-MF semi-confluent monolayer. The cells were incubated with 5 % CO_2_ at 37 °C within DMEM with 5 % FBS. The glass slides were collected 5 and 7 days after co-culture for morphological analysis of granulocytes eosinophils using May-Grünwald and Giemsa stains (Sigma Aldrich). For 3D co-culture, GR-MF in suspension were co-cultured with non-adherent mononuclear bone marrow cells of schistosome-infected C3H/HeN mice and seeded together in agarose 1 % coated round-bottom 96-well plates. Spheres were observed and collected at day 4 for histological analysis.

### Eosinophil peroxidase (EPO) detection assay

A colorimetric assay for eosinophil peroxidase was used to detect eosinophils in the co-culture of GR-MF with bone marrow cells. The substrate solution consists of 0.1 mM *o*-ophenylenediamine (OPD) in 0.05 M Tris–HCl buffer pH 8.0 containing 1 mM hydrogen peroxide, as described by Strath et al, 1985 [26]. The cell lysates were obtained by freezing, and total protein was normalized by BCA protein Kit.

### Statistical analysis

Statistical analysis was done using analysis of variance and the paired and unpaired Student *t* test. Results are expressed as mean ± SEM, and differences were considered significant at a *P* value < 0.05.

## Results

### Schistosome hepatic granulomas characterization

**Fig. 1:**
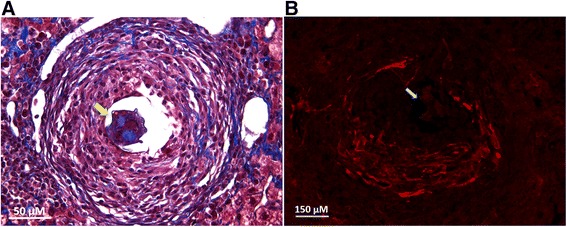
Histological analysis of hepatic granulomas **a** Representative photomicrograph of chronic schistosomal granulomas stained with Masson’s Trichrome Staining. **b** Immunofluorescence for α-actin in chronic granulomas. The parasite eggs are present in both granulomas and are indicated by *yellow arrow*. Magnification: **a** 1000x and **b** 400x. For histological analysis of normal liver, see Additional file 2: Figure S2

Schistosomiasis is characterized by an intense periovular granulomatous reaction, with activation of fibrogenic cells and collagen deposition. In our experimental model of the chronic phase of the disease, we observed an intense deposition of collagen within granulomas, as shown by Masson’s trichrome staining (Fig. 1a), as well the immunofluorescence for α-SMA (Fig. 1b), indicating GR-MF activation within granulomas.

### GR-MF characterization and activation in three-dimensional (3D) culture system

We detected myofibroblastic cells positive for smooth muscle α-actin around granulomas (Fig. 1b), potentially generated by activation and migration of adjacent perisinusoidal stellate cells and/or mobilization of circulating mesenchymal cell progenitors, and they were therefore termed here “granuloma myofibroblasts” (GR-MF).

**Fig. 2:**
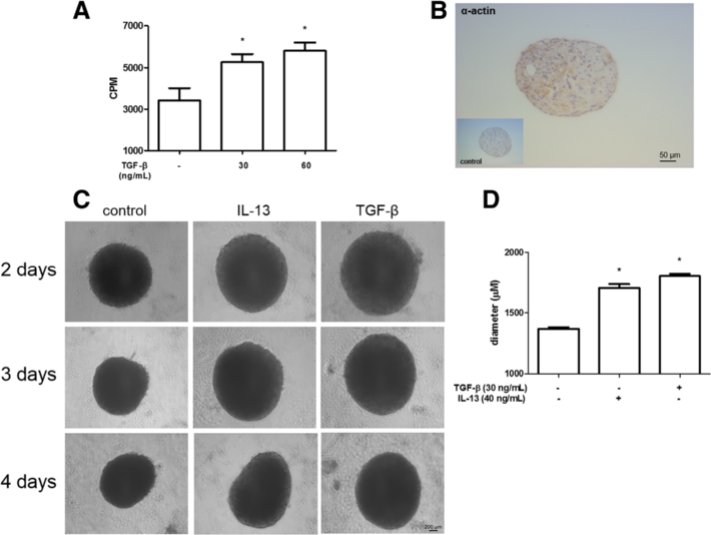
GR-MF spheroids stimulation by TGF-β and IL-13 **a** GR-MF were stimulated, in monolayer, with TGF-β (30 and 60 ng/mL) for 20 h. Cells were incubated with 3[H]-thymidine for 4 h. **b** Detection of α-actin by immunoenzymatic reaction in spheroids. **c** Representative photomicrography of GR-MF spheroids cultured for 4 days in the presence of TGF-β (30 ng/mL) or IL-13 (40 ng/mL). **d** Spheres diameters from at least four spheres from each group were measured using the Image Pro Plus program. This graph is representative from three experiments. *P ≤ 0.05 compared with non-stimulated cells

**Fig. 3:**
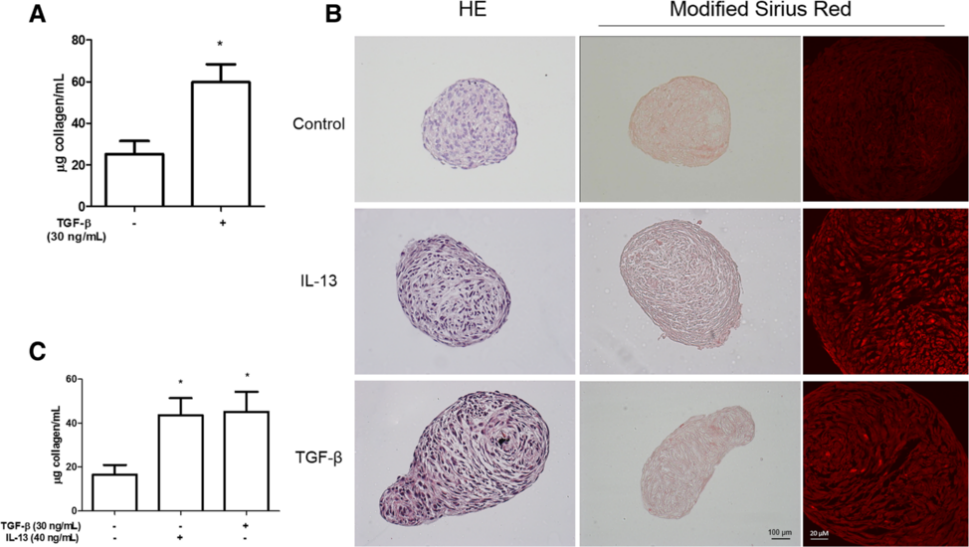
Collagen measurement and analysis of GR-MF spheroids **a** Collagen measurement into supernatants from non-stimulated or TGF-β (30 ng/mL) stimulated cells after 24 h of culture in monolayer **b** Representative photomicrographs of spheroids stained with haematoxylin/eosin (HE) and Modified Sirius Redstaining for collagen, optical microscope analyses (middle column) and confocal microscope analyses (right column). **c** Collagen measurement into supernatants from nonstimulated, TGF-β (30 ng/mL) or IL-13 (40 ng/mL) stimulated spheroids after 4 days of culture. Each bar represents the mean ± SEM, *n* = 3. This graph is representative from three experiments. *P ≤ 0.05 compared with non-stimulated cells

The role of TGF-β on activation, proliferation and migration of GR-MF has been well described, and confirmed in our *in vitro* model, as shown in Fig. 2a. TGF-β stimulates an increased synthesis of extracellular matrix proteins by connective tissue cells during granulomatous liver reaction [27]. In order to study a model closer to the structure and to the kinetics of myofibroblast cell activation within granulomas, we used a 3D culture system in spheroids, and reported that GR-MF retained their phenotype by immunohistochemistry for α-smooth muscle actin (Fig. 2b). We observed that GR-MF spheroids stimulated with TGF-β and IL-13 showed larger diameters than the untreated spheroids (Fig. 2c and d). These differences were observed between stimulated and untreated spheroids in all the days analyzed. We observed a significant decrease in the size of spheroids from the second to the 4th day in culture. These data confirm the capacity of GR-MF to respond to pro-inflammatory stimuli as observed previously in the 2D system. While we observed that TGF-β stimulates GR-MF proliferation (Fig. 2a), we also reported an increased secretion of collagen into the supernatant of stimulated cells (Fig. 3a). Histological analysis of GR-MF spheroids also illustrated the degree of cell activation. We observed that the arrangement of the cells, by HE staining, and collagen fibers, by modified Sirius red staining (optical and confocal microscope analyses), from spheroids stimulated with TGF-β and IL-13 are more concentric and elongated when compared to non-stimulated spheroids (Fig. 3b). We also observed an increased amount of collagen in the supernatant of GR-MF spheroids stimulated with TGF-β and IL-13 (Fig. 3c). Therefore, these results show that GR-MF upon pro-fibrotic stimulation behave differently from non-stimulated cells.

### IL-5 and eotaxin detection in GR-MF

**Fig. 4:**
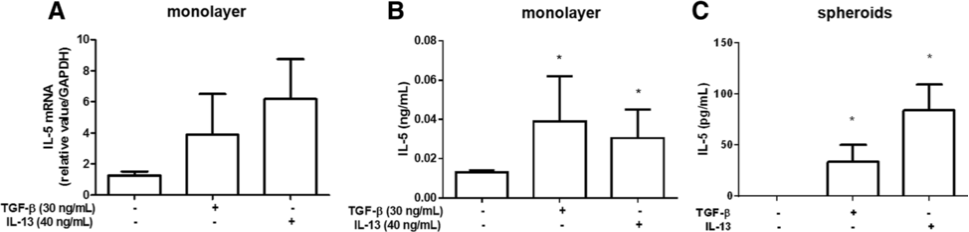
IL-5 expression by GR-MF **a** Real time RT-PCR to determine the relative expression of IL-5 by GR-MF stimulated with TGF-β (30 ng/mL) and IL-13 (40 ng/mL). The relative value was obtained in relation to GAPDH expression. **b** IL-5 levels measured by ELISA into supernatant of GR-MF stimulated with TGF-β (30 ng/mL) and IL-13 (40 ng/mL). **c** IL-5 levels measured by ELISA into supernatant of GR-MF spheroids stimulated with TGF-β (30 ng/mL) and IL-13 (40 ng/mL) after 4 days in culture. These graphs are representative from three experiments. *P ≤ 0.05 compared with non-stimulated cells

**Fig. 5:**
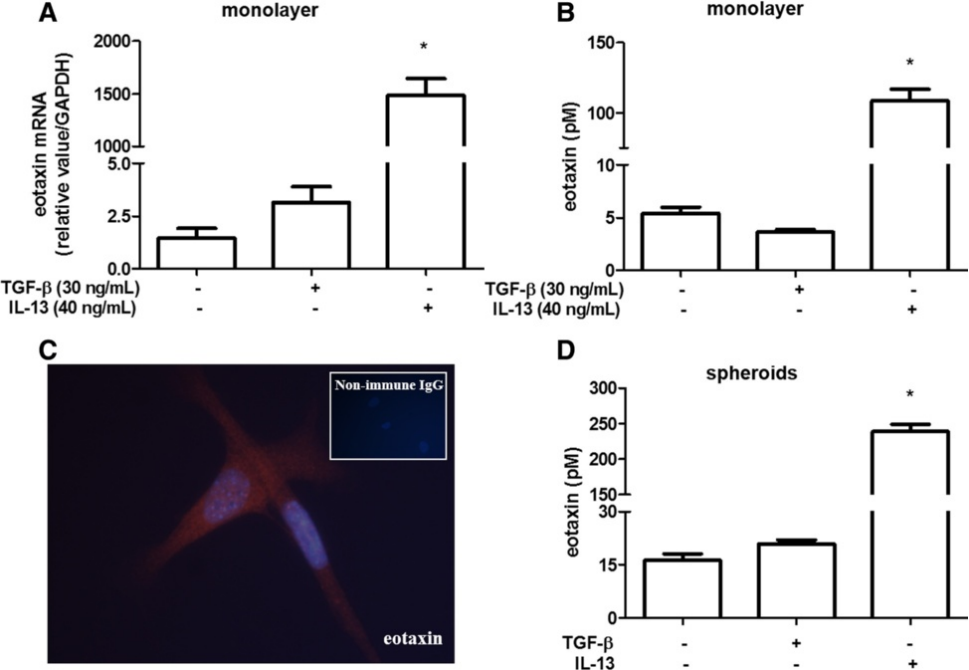
Eotaxin expression by GR-MF **a** Real time RT-PCR to determine the relative expression of eotaxin by GR-MF stimulated with TGF-β (30 ng/mL) and IL-13 (40 ng/mL). The relative value was obtained in relation to GAPDH expression. **b** Eotaxin levels measured by ELISA into supernatant of GR-MF stimulated with TGF-β (30 ng/mL) and IL-13 (40 ng/mL). **c** Immunolocalization of eotaxin in GR-MF. Magnification 600X. **d** Eotaxin levels measured by ELISA into supernatant of GR-MF spheroids stimulated with TGF-β (30 ng/mL) and IL-13 (40 ng/mL) after 4 days in culture. These graphs are representative of three experiments. *P ≤ 0.05 compared with non-stimulated cells

During infection by *S. mansoni*, there is an intense extramedullar proliferation of hematopoietic cells, mainly eosinophils, in hepatic granuloma [10, 16]. Furthermore, studies support the hypothesis that the GR-MF have similar characteristics of the hematopoietic bone marrow stroma, and are able to sustain myelopoiesis both *in vivo* and *in vitro* [17]. Therefore, to investigate whether GR-MF are able to produce and secrete cytokines important in eosinophilia, we evaluated the expression of IL-5 and eotaxin, two relevant cytokines that control eosinophils. We demonstrated that GR-MF can produce IL-5 spontaneously *in vitro*, as shown by mRNA detection (Fig. 4a) and by ELISA in the supernatant of these cultured cells (Fig. 4b). When cells were stimulated with TGF-β and IL-13, we detected an enhanced IL-5 mRNA and protein production by treated cells. The same phenomenon was observed when IL-5 was measured into supernatant of stimulated spheroids (Fig. 4c). Some studies have demonstrated that eotaxin, may be associated with extracellular matrix proteoglycans [28]. While myofibroblasts are involved in extracellular matrix production within granulomas, we considered that they could be also an important source of the eotaxin production. Under normal culture conditions, GR-MF express eotaxin mRNA (Fig. 5a) and secrete basal levels of this cytokine into their supernatant (Fig. 5b). We also could detect by immunofluorescence the expression of eotaxin *in vitro* by non-stimulated cells (Fig. 5c). While both TGF-β and IL-13 stimulation induced production of IL-5, only the stimulation with IL-13 was able to induce a significant increase in the expression of eotaxin mRNA and in its secretion by GR-MF. Similar data were also observed in the more complex 3D system of GR-MF culture (Fig. 5d). These data indicate that the production of cytokines and chemokines by GR-MF is a highly regulated and stimulus-specific phenomenon.

### Eosinophils in GR-MF microenvironment

We have reported that the GR-MF are cells with high pro-inflammatory potential, through production of leukotrienes, cytokines and chemokines [13]. Our data show that GR-MF express relevant stimuli for eosinophils, IL-5 and eotaxin, under stimulation with fibrogenic stimuli. Further, we have investigated the capacity of cultured-GR-MF to sustain eosinophils *in vitro*.

**Fig. 6:**
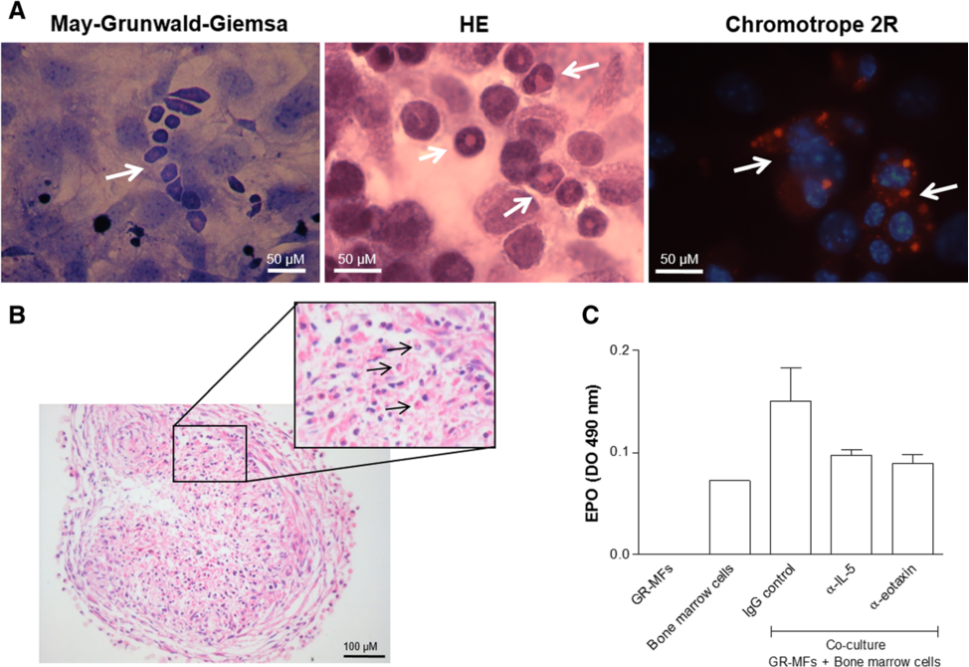
Co-culture of GR-MF with non-adherent hematopoietc bone marrow cells obtained from mice infected with *S. mansoni* in the chronic phase **a** Clusters of eosinophils observed in monalayer co-culture, after 7 days, stained with May-Grünwald/Giemsa, Hematoxylin/Eosin and Chromotrope 2R. **b** Eosinophils (*arrows*) observed in spheroids co-culture, after 4 days, stained with Hematoxilin/Eosin. **c** Measurement of EPO levels in co-culture treated with neutralizing antibodies for IL-5 and eotaxin. This graph is representative from three experiments. *P ≤ 0.05 compared with non-treated cells

By employing co-cultures of GR-MF with non-adherent cells from bone marrow, in both monolayer and spheroids models, we have observed that GR-MF were able to sustain eosinophil population *in vitro*. We observed the formation of eosinophilic clusters within 7 days in co-cultures of GR-MF monolayer (Fig. 6a), and after 3 days in co-culture with GR-MF spheroids (Fig. 6b). Eosinophilic clusters were mostly detected on GR-MF monolayers when co-cultured with hematopoietic cells from schistosome-infected mice rich in eosinophil precursors.

In order to better elucidate the mechanisms involved in the ability of GR-MF monolayers to control eosinophil populations observed in co-cultures, we evaluated the role of GR-MF-derived IL-5 and eotaxin. Co-cultured GR-MF monolayers and hematopoietic cells from schistosome-infected mice were treated with neutralizing monoclonal antibodies against IL-5 or eotaxin. In order to monitor the eosinophil granulocyte population, after 5 days of co-cultures cells were disrupted and eosinophil peroxidase (an eosinophil protein marker) was measured in the cell lysate. Figure 6c shows that neutralization of IL-5 and eotaxin by monoclonal antibodies promotes a clear decrease of EPO levels in co-culture. These data suggest that the neutralization of GR-MF-derived IL-5 and eotaxin reduces GR-MF capacity to maintain eosinophil population in the co-culture and are in agreement with previous results showing that extra-medullary eosinopoiesis occurs within *S. mansoni* infection-induced liver granuloma [8]. Altogether, we can conclude that the GR-MF have an important role in the eosinophilia observed in liver granuloma during infection.

## Discussion

In the experimental model of *S. mansoni* infection, we observed that myofibroblasts obtained from schistosome granulomas are able to respond to TGF-β and IL-13 stimuli *in vitro*, increasing the rate of proliferation and collagen production. In order to better evaluate the activation and the role of GR-MF during infection, we used a three-dimensional model of cell culture in spheroids. The three-dimensional models are better than conventional cultures, since they promote cell–cell and cell–extracellular matrix contacts that are necessary for many cellular function such as differentiation, proliferation, cell death, apoptosis and angiogenesis [29]. We observed that the GR-MF are able to form spheroids, keeping their myofibroblast phenotype.

In order to identify an immunoregulatory role of these cells during formation of hepatic granulomas, we analyzed the production of IL-5 and eotaxin, important mediators involved in the proliferation and differentiation of eosinophils [30]. In our experiments, we detect by real-time PCR and ELISA assays, that GR-MF express and secrete IL-5 and eotaxin. When cells were stimulated with TGF-β and IL-13, an increased production of IL-5 was observed, while IL-13, but not TGF- β, stimulated eotaxin production. Pro-fibrogenic cytokines such as IL-13 and TGF-β are able to stimulate cells in 3D spheroids, triggering an increase in diameter, in collagen production and secretion of IL-5 and eotaxin in the supernatant. When performing the histological analysis of spheroids, we observed that stimulated spheroids have a completely different arrangement of cells and collagen fibers, reflecting the activation of these cells induced by IL-13 and TGF-β.

During fibrosis progression, hepatocellular damage and inflammation trigger complex cellular events, including myofibroblasts activation that result in collagen deposition and the disruption of the normal liver architecture [19]. Recent studies have shown that hepatic myofibroblasts have origin from a resident stellate cells in the liver parenchyma [31]. Their activation as a hallmark during the development of periovular granulomas [32].

Besides their role in fibrosis, activated stellate cells participate in various immunomodulatory events, amplifying the inflammatory response through the production of chemokines, such as MCP-1 and RANTES, expression of chemokine receptors, as CCR5, and could behave as antigen presenting cells [33, 34]. Bacterial endotoxin also stimulates stellate cells through alteration in expression of numerous relevant genes, such as the down regulation of TGF-β1 [20]. They were also described as bystander in the activation of Tregs, due to their ability to produce and store retinoic acid into lipid bodies, that is important to promote TGF-β-dependent Foxp3+ Treg induction and suppression of Th17 differentiation [35].

In the murine model of schistosomiasis, myofibroblasts derived from hepatic granulomas have also an immunoregulatory function expressing and producing a number of proinflammatory cytokines such as GM-CSF and SCF, related to liver tissue repair and sustaining the development of inflammation [10, 18, 36]. It was also reported that granuloma myofibroblasts are able to release cysteinyl-leukotrienes in a TGF-β regulated manner contributing to the pathogenesis and tissue repair in schistosomiasis [21].

During chronic *S. mansoni* infection, the number of eosinophil precursors increases in the bone marrow and in extramedullary myelopoietic foci [10, 37] due not only to bone marrow production but, similarly to a monomacrophagic population, and to their amplification in peripheral tissues [8]. It is well established that leukocyte interactions within stromal cells and endothelial cells are essential for its recruitment into inflamed tissues [38].

Considering the production of IL-5 and eotaxin by activated GR-MF, we monitored the ability of GR-MF to promote eosinophil survival when co-cultured *in vitro* with non-adherent bone marrow cells from *S. mansoni* infected mice. We also observed the presence of eosinophils in multicellular spheroids of GR-MF containing non-adherent bone marrow cells from *S. mansoni* infected mice. When we use neutralizing antibodies to block IL-5 and eotaxin in the co-culture, we observed a low EPO activity in the co-cultures indicating that these cytokines produced by GR-MF are important for eosinophil survival. Together, these data suggest an important role of GR-MF for eosinophil recruitment and survival in periovular granulomas.

## Conclusion

The present research shows that GR-MF derived from periovular granulomas of *S. mansoni*-infected mice produce IL-5 and eotaxin under TGF-β and IL-13 stimulation, and provides evidence that these molecules are relevant for eosinophil production and maintenance in hepatic granulomas.

## Additional files

Additional file 1: Figure S1. Characterization of GR-MFs after the fourth passage in cell culture. (A) GR-MFs stained for toluidine blue. Observe a stained mast cell from peritoneal fluid inset. (B) GR-MFs stained for Sirius red, with modified pH 10.2. Observe stained eosinophils from bone marrow inset. (C) GR-MFs stained for α-naphthyl esterase. Observe stained macrophages from bone marrow inset. (D) GR-MFs immunolocalization of α-SM actin. Magnification (A–B) 400X and (C–D) 600X. (TIF 2494 kb) Additional file 2: Figure S2. Histological analysis of normal liver (A) Representative photomicrograph of normal liver stained with *Masson’s* Trichrome *Staining.* (B) Immunofluorescence for α-actin in normal liver*.* We could observe a blood vessel in the left side of each image. Magnification: (A) 1000x and (B) 400x. (TIF 2526 kb)
