# Supplementary figures and images for: Hepatic myofibroblasts derived from Schistosoma mansoni-infected mice are a source of IL-5 and eotaxin: controls of eosinophil populations in vitro
Source: Parasit Vectors. 2015 Nov 9;8:577. doi: 10.1186/s13071-015-1197-3 (PMC4640404; doi:10.1186/s13071-015-1197-3)

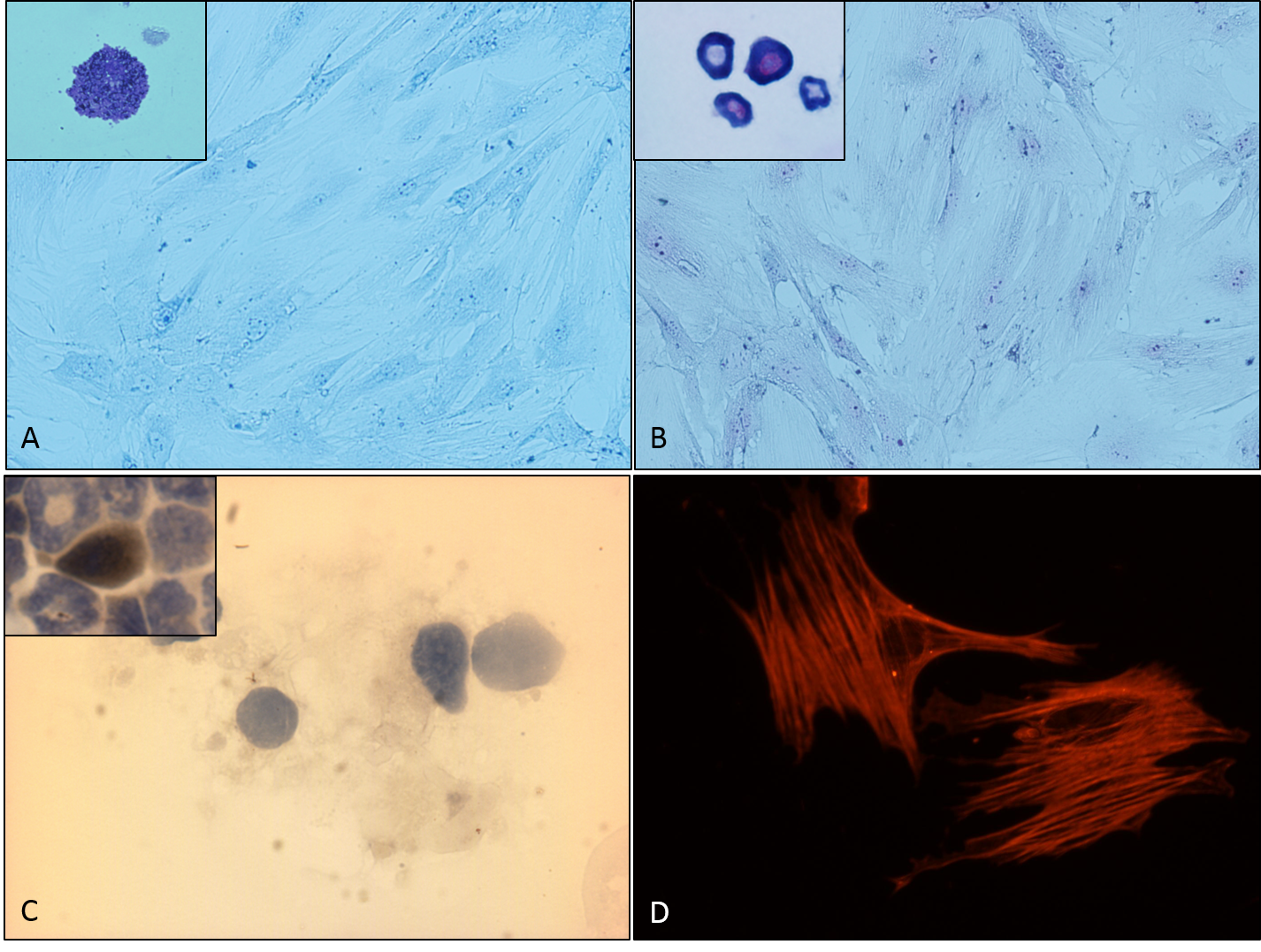

Supplement: Additional file 1: Figure S1. — Characterization of GR-MFs after the fourth passage in cell culture. (A) GR-MFs stained for toluidine blue. Observe a stained mast cell from peritoneal fluid inset. (B) GR-MFs stained for Sirius red, with modified pH 10.2. Observe stained eosinophils from bone marrow inset. (C) GR-MFs stained for α-naphthyl esterase. Observe stained macrophages from bone marrow inset. (D) GR-MFs immunolocalization of α-SM actin. Magnification (A–B) 400X and (C–D) 600X. (TIF 2494 kb) [file 13071_2015_1197_MOESM1_ESM.tif]

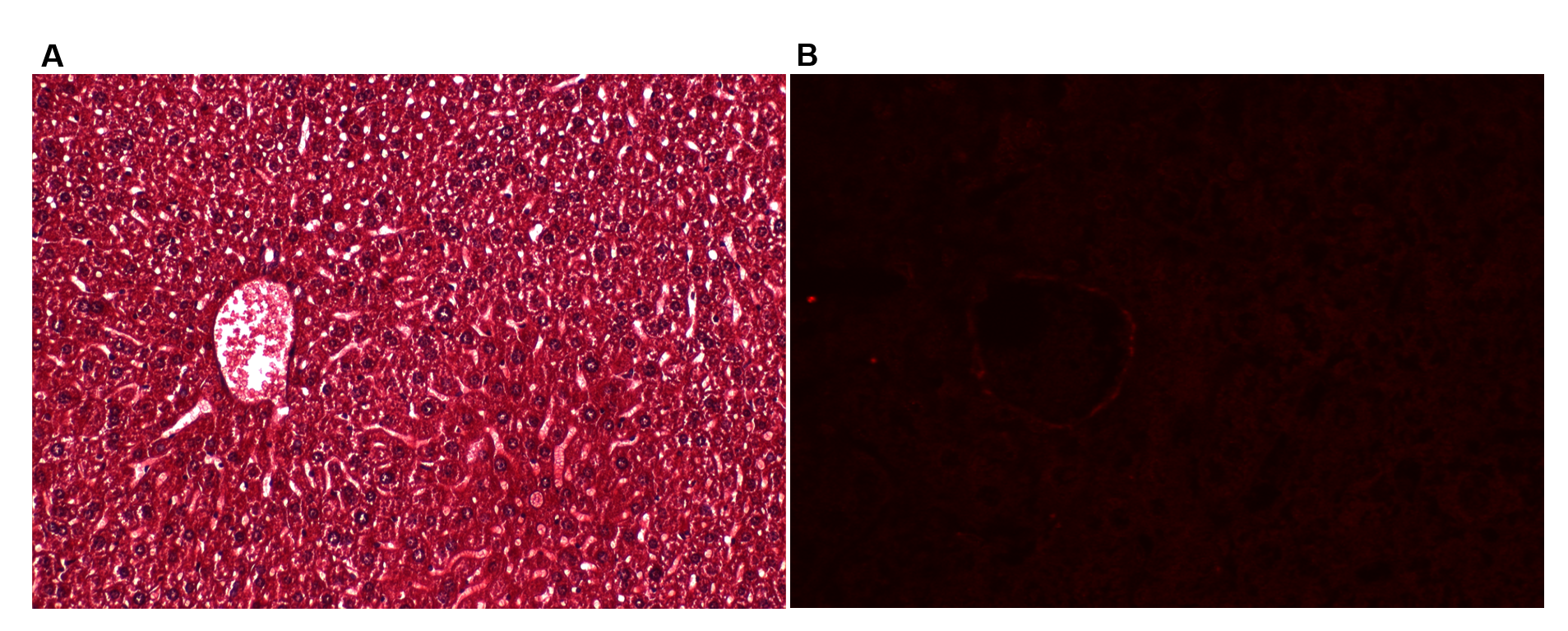

Supplement: Additional file 2: Figure S2. — Histological analysis of normal liver (A) Representative photomicrograph of normal liver stained with Masson’s Trichrome Staining. (B) Immunofluorescence for α-actin in normal liver. We could observe a blood vessel in the left side of each image. Magnification: (A) 1000x and (B) 400x. (TIF 2526 kb) [file 13071_2015_1197_MOESM2_ESM.tif]
